# Experimental Determination of the p*K*_a_ Values of Clinically Relevant Aminoglycoside Antibiotics: Toward Establishing p*K*_a_—Activity Relationships

**DOI:** 10.1021/acsomega.3c09226

**Published:** 2024-01-26

**Authors:** Ruslans Muhamadejevs, Klara Haldimann, Marina Gysin, David Crich, Kristaps Jaudzems, Sven N. Hobbie

**Affiliations:** † Latvian Institute of Organic Synthesis, Aizkraukles 21, LV-1006 Riga, Latvia; ‡ Universität Zürich, Institute of Medical Microbiology, Gloriastrasse 30, CH-8006 Zürich, Switzerland; § Department of Pharmaceutical and Biomedical Sciences, University of Georgia, 250 West Green Street, Athens, Georgia 30602, United States; ∥ Division of Clinical Bacteriology and Mycology, University Hospital Basel, 4031 Basel, Switzerland

## Abstract

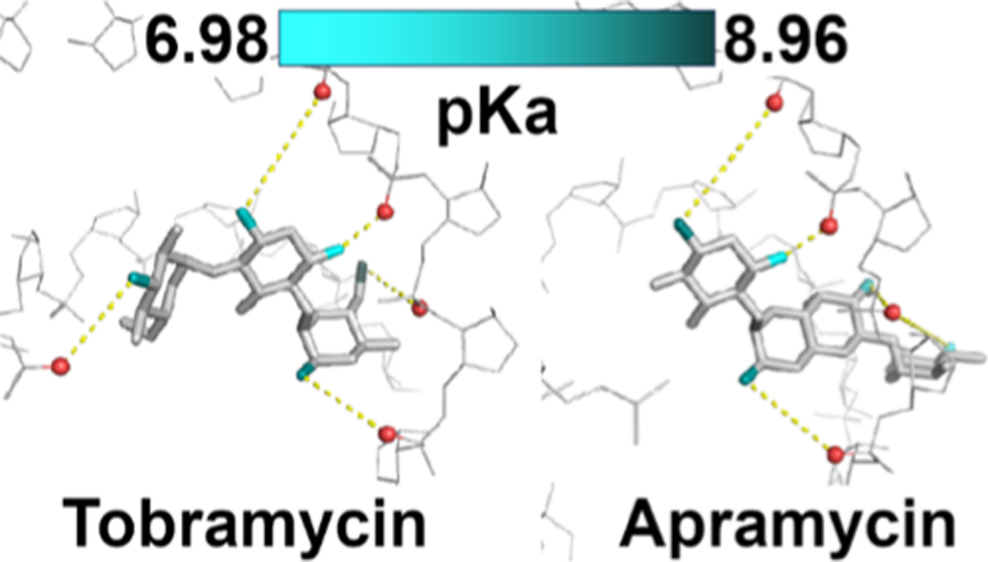

Investigating the relationship between individual p*K*_a_ values and the efficacy of aminoglycosides is essential for the development of more effective and targeted therapies. In this work, we measured the p*K*_a_ values for individual amino groups of the six clinically relevant aminoglycoside antibiotics gentamicin, tobramycin, amikacin, arbekacin, plazomicin, and apramycin using ^15^N–^1^H heteronuclear multiple-bond correlation and ^1^H NMR experiments. For arbekacin and plazomicin, the p*K*_a_ values are reported for the first time. These p*K*_a_ values were used to calculate the net charges of the aminoglycosides and the protonation levels of amino groups under various pH conditions. The results were analyzed in relation to the mode of interaction and inhibition to establish p*K*_a_ relationships for rRNA binding, inhibitory activity, and the pH dependence of the uptake into bacterial cells.

## Introduction

Aminoglycosides are a class of natural antibiotics derived from various species of *Streptomyces* and *Micromonospora* bacteria and used primarily in the treatment of severe Gram-negative and mycobacterial infections.^1,2^ They are structurally complex molecules composed of an aminocyclitol moiety and amino sugars connected by glycosidic linkages. Based on the identity of the aminocyclitol, the aminoglycosides can be divided into five groups: (1) derivatives containing streptidine (e.g., streptomycin), (2) those containing streptamine (spectinomycin), (3) those containing a 4,5-disubstituted deoxystreptamine (neomycin), and (4) derivatives containing a 4,6-disubstituted deoxystreptamine moiety (gentamicin, kanamycin, tobramycin, and sisomicin). Apramycin constitutes a fifth group that is structurally unique in that it contains a 4-monosubstituted deoxystreptamine aminocyclitol moiety and a bicyclic sugar moiety. Its unique chemical structure protects apramycin from almost all the bacterial resistance mechanisms that inactivate all other aminoglycoside antibiotics in clinical use, spurring interest in apramycin as a potential drug candidate.^4−6^ In addition, several semisynthetic aminoglycoside analogues are available bearing a variety of amino and/or hydroxyl substitutions (e.g., amikacin, arbekacin, and plazomicin). These derivatives are used for the treatment of infections caused by multiresistant bacteria as the introduced groups can influence the mechanism of action and susceptibility to various aminoglycoside modifying enzymes.^7,8^

The aminoglycoside antibiotics act by binding to the 16S ribosomal RNA (rRNA) in the 30S ribosomal subunit, impeding the fidelity of the translation process.^9−11^ This interference results in the disruption of the bacterial protein synthesis, ultimately leading to cell death. The high-affinity binding to rRNA varies among the distinct classes of aminoglycosides; however, in all cases, it is in part driven by electrostatic interactions between the positively charged aminoglycoside ammonium groups and the negatively charged sugar–phosphate backbone of rRNA.^12,13^ Additionally, the positive charge facilitates aminoglycoside binding to the negatively charged bacterial cell surface and allows them to penetrate through the outer membrane.^14^ This implies that the ionization constants (p*K*_a_s) and locations of the amino groups in the aminoglycoside structure can have a significant impact on the biological activity of these antibiotics.

Understanding structure–activity relationships of aminoglycosides requires knowing the protonation level of individual amino groups at a specific pH found at the site of infection. Traditional ^15^N NMR titration methods have been used to measure the site-specific p*K*_a_ values of amino groups in various aminoglycosides, including neomycin B,^15^ apramycin,^16,17^ isepamicin,^18^ ribostamycin,^19^ and tobramycin.^16,20^ However, minor differences between the equipment and experimental approaches used in these separate studies complicates a direct comparison of the aminoglycosides in terms of ionization state at a particular pH. Recently, Blagbrough and co-workers published two studies reporting individual p*K*_a_ values for a total of nine selected aminoglycosides from *Streptomyces* and *Micromonospora* (tobramycin, kanamycin B, amikacin, sisomicin, netilmicin, neamine, neomycin, paromomycin, and streptomycin) by multinuclear NMR spectroscopy.^21,22^ The determined p*K*_a_ values deviated from previously published data by up to 1.5 units, highlighting the impact of different experimental protocols and calling for more comparative studies. Notably, such differences would preclude the establishment of meaningful structure–activity relationships.

In this work, we determine the p*K*_a_ values for individual amino groups of apramycin in comparison to the five clinically most relevant aminoglycoside antibiotics using ^15^N–^1^H heteronuclear multiple-bond correlation (HMBC) and ^1^H NMR titration experiments. Based on the p*K*_a_ values, we calculate the pH dependent net charge of the aminoglycosides and the protonation levels of amino groups. Furthermore, we analyze the structure—p*K*_a_ relationships and protonation profiles of the aminoglycosides in relation to their reported rRNA-bound structures and uptake from different biological environments. Our findings demonstrate that this approach allows for the identification of the amino groups that contribute most to binding and ribosomal inhibition at specific pH levels.

## Results and Discussion

The p*K*_a_ values of individual amino groups of the studied aminoglycosides were determined using a similar approach as described by Alkhzem et al.^21^ In short, the aminoglycoside samples were dissolved in D_2_O and titrated with DCl or NaOD. Changes in the ^15^N and ^1^H chemical shifts were monitored by using ^15^N–^1^H HMBC and ^1^H NMR spectroscopy. Assignment of the ^1^H, ^13^C, and ^15^N resonances was performed based on 2D correlation spectra. The p*K*_a_s were calculated using Microsoft Excel Solver Add-in by fitting the experimental data to a theoretical model (see the Experimental Section for details). Spectroscopic measurements were repeated twice to obtain data points in triplicate for each aminoglycoside.

### Gentamicin

**Figure 1:**
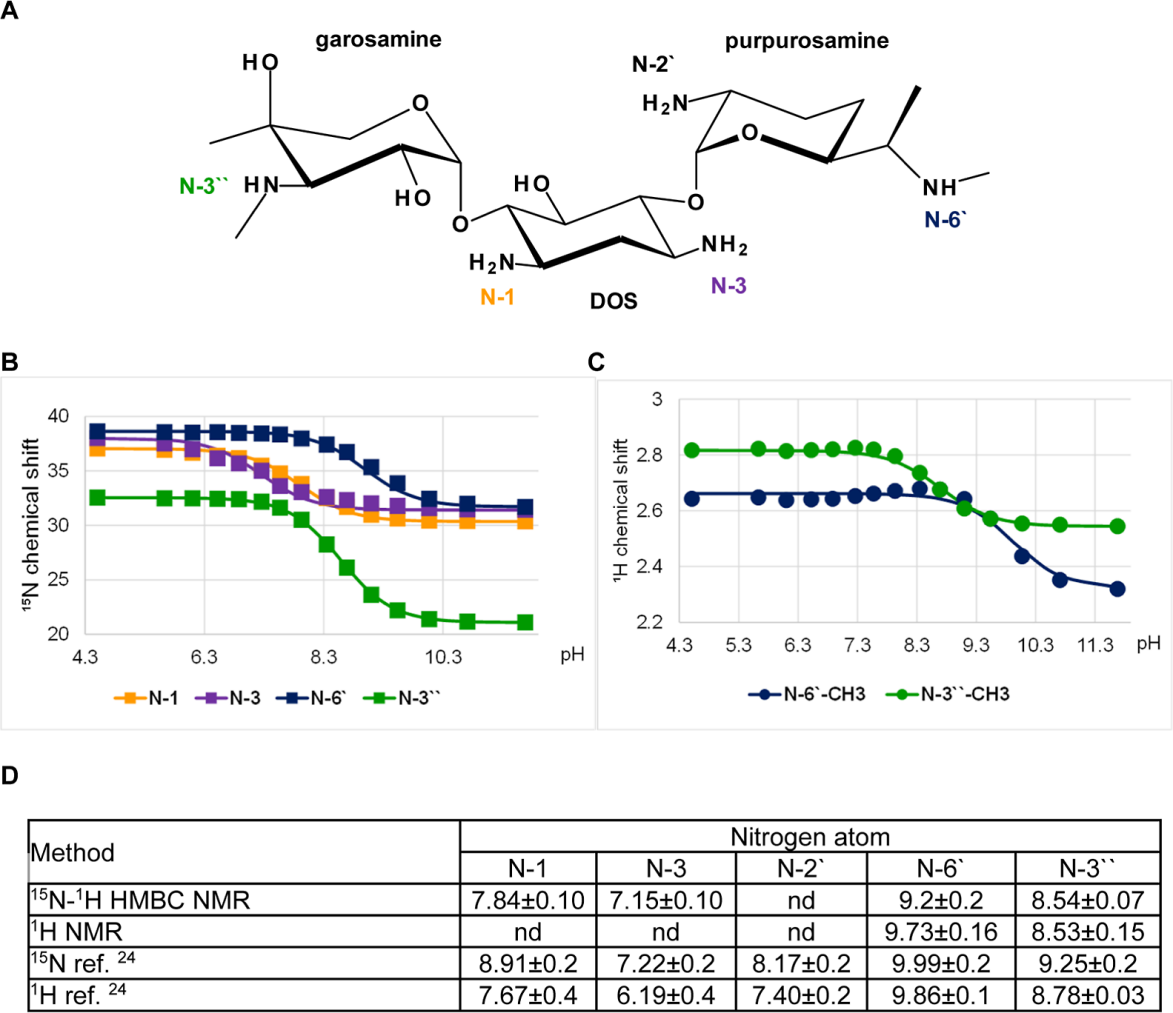
p*K*_a_ determination for individual amino groups of gentamicin. (A) Chemical structure and atom numbering of gentamicin. (B) ^15^N NMR chemical shift titration curves. (C) ^1^H NMR chemical shift titration curves. (D) Determined p*K*_a_ values in comparison to values published previously.

Gentamicin is a 4,6-disubstituted deoxystreptamine derivative containing purpurosamine at the 4-O and garosamine (3-methyl amino-3-deoxy-4-*C*-methyl-β-l-arabinose) at the 6-O positions (Figure 1A). Naturally produced gentamicin is a mixture of five major compounds C1, C1a, C2, C2a, and C2b differing by the substituents at the 6′ position of the purpurosamine ring.^23^ Gentamicin C1, C2a, and C2b have three primary amine (i.e., monosubstituted ammonia) groups, N-1 and N-3 on the DOS moiety and N-2′ on purpurosamine; as well as two secondary amines, N-6′ on purpurosamine and N-3′′ on garosamine. In gentamicin C1a and C2, N-6′ is a primary amine bringing the balance to four primary and one secondary amine instead. Correlation with N-2′ was not observed by ^15^N–^1^H HMBC NMR and the H-1, H-3, and H-2′ chemical shifts could not be assigned in the ^1^H NMR spectra due to spectral overlap, which probably stems from the fact that the sample is a mixture of congeners. Therefore, the p*K*_a_s for N-6′, N-3′′ were measured using ^15^N and ^1^H chemical shift data, whereas for N-1 and N-3, the p*K*_a_s were measured using only ^15^N chemical shift data (Figure 1B,C). The ^1^H and ^15^N derived values are in good agreement for N-3′′ but differ by ∼0.6 units for N-6′. The order of the p*K*_a_ values is N-6′ > N-3′′ > N-1 > N-3, which is consistent with literature data (Figure 1D).^24^ However, our determined p*K*_a_ values are about 0.1–1.1 units lower when considering the ^15^N NMR data and by 0.1–0.3 units lower when considering the ^1^H NMR data. The reasons for these discrepancies may be incorrect assignments due to the spectral overlap or very different sample concentrations used for the ^15^N and ^1^H NMR measurements, respectively, as noted by previous authors.^24^

### Tobramycin

**Figure 2:**
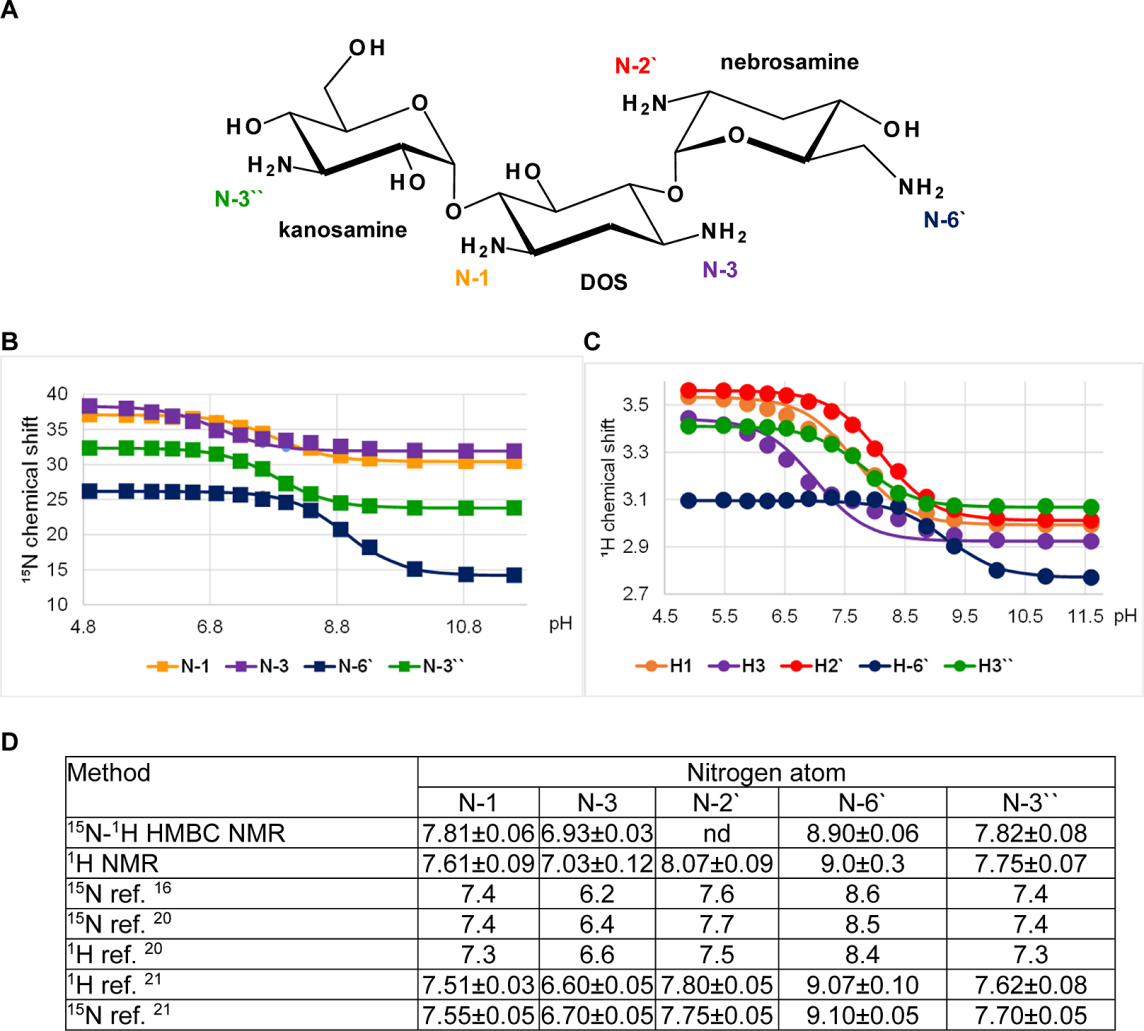
p*K*_a_ determination for the individual amino groups of tobramycin. (A) Chemical structure and atom numbering of tobramycin. (B) ^15^N NMR chemical shift titration curves. (C) ^1^H NMR chemical shift titration curves. (D) Determined p*K*_a_ values in comparison to values published previously.

Tobramycin is a 4,6-disubstituted deoxystreptamine derivative containing nebrosamine at the 4-O and 3-amino-3-deoxy-d-glucose (kanosamine) at the 6-O positions (Figure 2A). The kanosamine moiety is present in several aminoglycosides (e.g., tobramycin, kanamycin, and amikacin) and is an antibiotic by itself.^25^ Tobramycin has five primary amine groups, N-1 and N-3 on DOS, N-2′ and N-6′ on nebrosamine, and N-3′′ on kanosamine. Again, the N-2′ atom was not observed by ^15^N–^1^H HMBC NMR and its p*K*_a_ value was determined from the H-2′ chemical shift data (Figure 2B,C). The p*K*_a_s determined from ^15^N and ^1^H chemical shift data agree well, except for N-1 where the difference is 0.05 units taking into account the uncertainty. The order of the p*K*_a_ values is N-6′ > N2′ > N-3′′ ≈ N-1 > N-3, which is consistent with literature data (Figure 2D).^16,20,21^ Our determined p*K*_a_ values differ by about 0.1–0.3 units compared to the latest report in the literature and by 0.3–0.7 units compared to earlier studies.

### Amikacin

**Figure 3:**
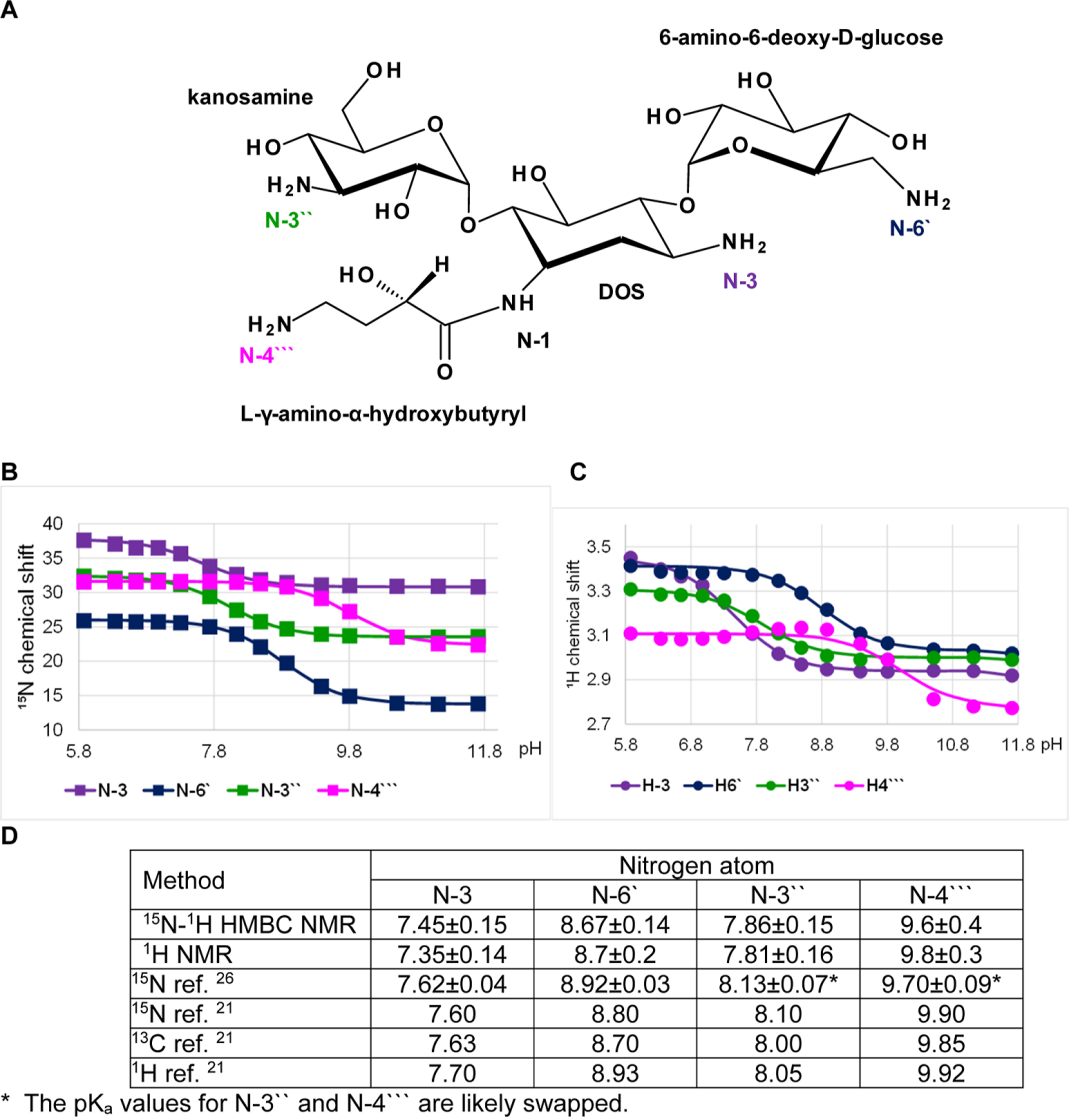
p*K*_a_ determination for the individual amino groups of amikacin. (A) Chemical structure and atom numbering of amikacin. (B) ^15^N NMR chemical shift titration curves. (C) ^1^H NMR chemical shift titration curves. (D) Determined p*K*_a_ values in comparison to values published previously.

Amikacin is a semisynthetic derivative of kanamycin. It consists of a 4,6-disubstituted deoxystreptamine with 6-amino-6-deoxy-d-glucose at the 4-O and kanosamine at the 6-O positions (Figure 3A). The DOS moiety is acylated with an l-γ-amino-α-hydroxybutyryl side chain at the 1-amino group. Amikacin has four primary amine groups, N-3 and N-4′′′ on DOS, N-6′ on 6-amino-6-deoxy-d-glucose, and N-3′′ on kanosamine. The p*K*_a_ values were determined from ^15^N and ^1^H chemical shift data for all amines and showed very good agreement (Figure 3B,C). The order (N-4′′′ > N-6′ > N-3′′ > N-3) and magnitude of the p*K*_a_ values is in good agreement with those reported in the literature (Figure 3D).^21,26^

### Arbekacin

**Figure 4:**
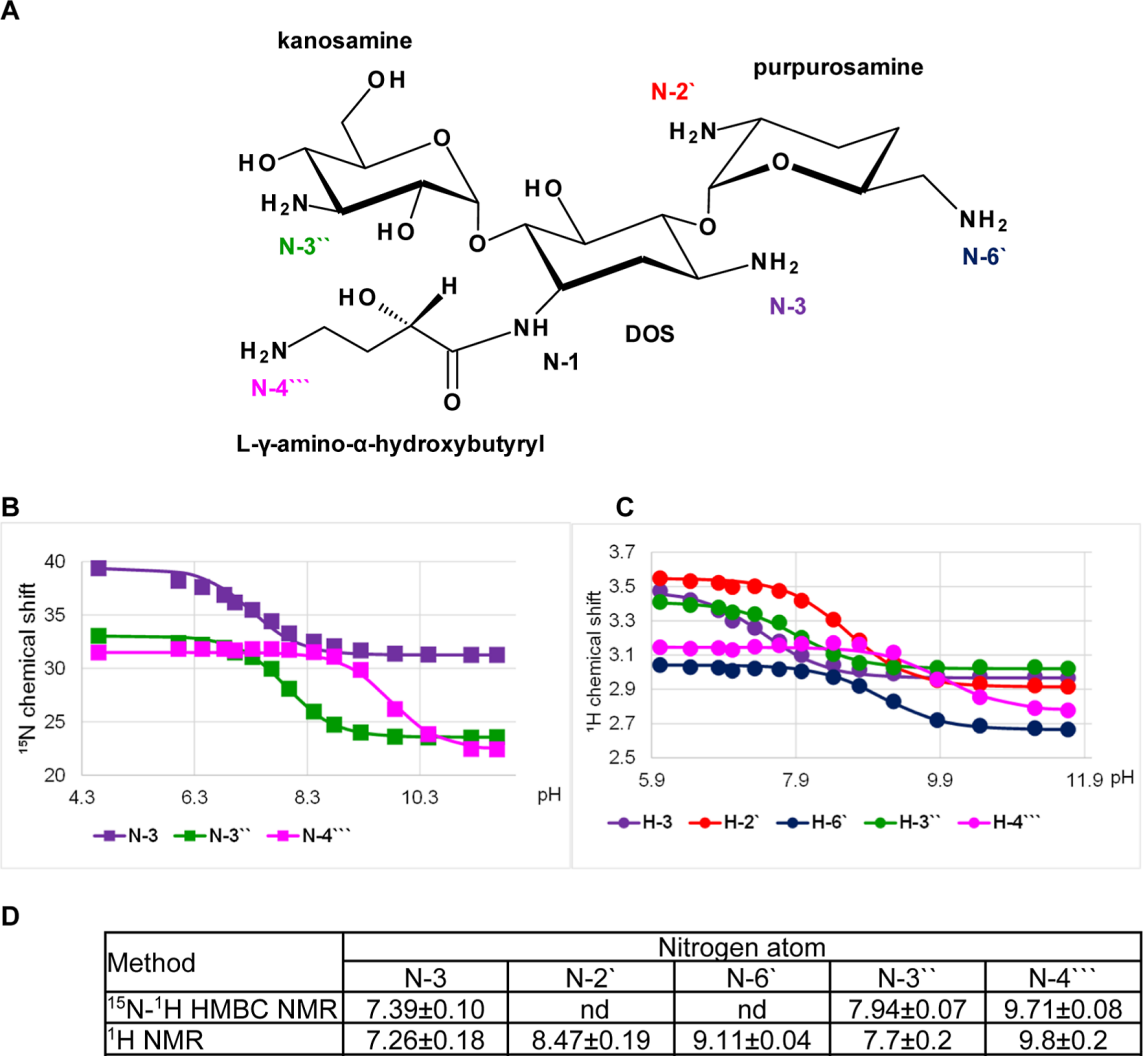
p*K*_a_ determination for the individual amino groups of arbekacin. (A) Chemical structure and atom numbering of arbekacin. (B) ^15^N NMR chemical shift titration curves. (C) ^1^H NMR chemical shift titration curves. (D) Determined p*K*_a_ values.

Arbekacin is another semisynthetic derivative of dibekacin (3′,4′-dideoxy-kanamycin). It contains a 4,6-disubstituted deoxystreptamine with purpurosamine C at the 4-O and kanosamine at the 6-O positions (Figure 4A). The DOS moiety is also acylated with an l-γ-amino-α-hydroxybutyryl side chain at the 1-amino group. It has five primary amine groups, N-3 and N-4′′′ on DOS, N-6′ on purpurosamine C, and N-3′′ on kanosamine. The p*K*_a_ values of N-3, N-3′′, and N-4′′′ amines were determined using both ^15^N and ^1^H chemical shift data and showed good agreement, while p*K*_a_ values of N-2′ and N-6′ were determined only from ^1^H chemical shifts (Figure 4B–D). The order of the p*K*_a_ values is N-4′′′ > N-6′ > N-3′′ > N-3 and is reported here for the first time.

### Plazomicin

**Figure 5:**
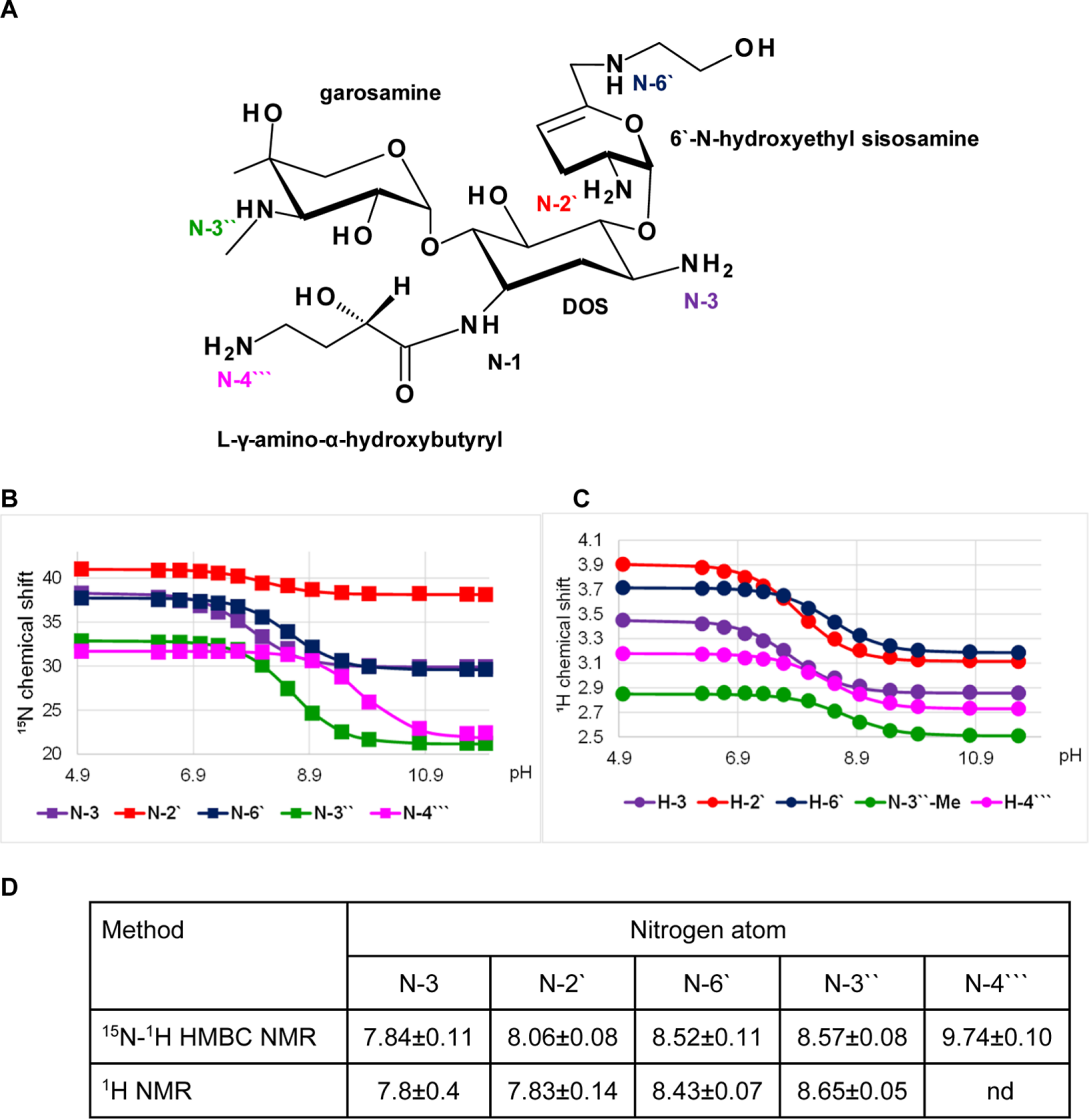
p*K*_a_ determination for the individual amino groups of plazomicin. (A) Chemical structure and atom numbering of plazomicin. (B) ^15^N NMR chemical shift titration curves. (C) ^1^H NMR chemical shift titration curves. (D) Determined p*K*_a_ values.

Plazomicin is a semisynthetic aminoglycoside derived from sisomicin. It has a 4,6-disubstituted deoxystreptamine with 6′-*N*-hydroxyethyl sisosamine at the 4-O and garosamine at the 6-O positions (Figure 5A). Similarly to amikacin and arbekacin, the DOS moiety is acylated with an l-γ-amino-α-hydroxybutyryl side chain at the 1-amino group. Plazomicin has three primary amine groups, N-3 and N-4′′′ on DOS, N-2′ on sisosamine, and two secondary amine groups, N-6′ on sisosamine and N-3′′ on garosamine. The p*K*_a_ values of N-3, N-2′, N-6′, and N-3′′ amines were determined from ^15^N and ^1^H chemical shift data and showed a good match, whereas N-4′′′ amine p*K*_a_s were determined using ^1^H and ^15^N NMR data, respectively (Figure 5B,C). The individual p*K*_a_ values and their order (N-4′′′ > N-3′′ ≈ N-6′ > N-2′ ≈ N-3) are reported here for the first time.

### Apramycin

**Figure 6:**
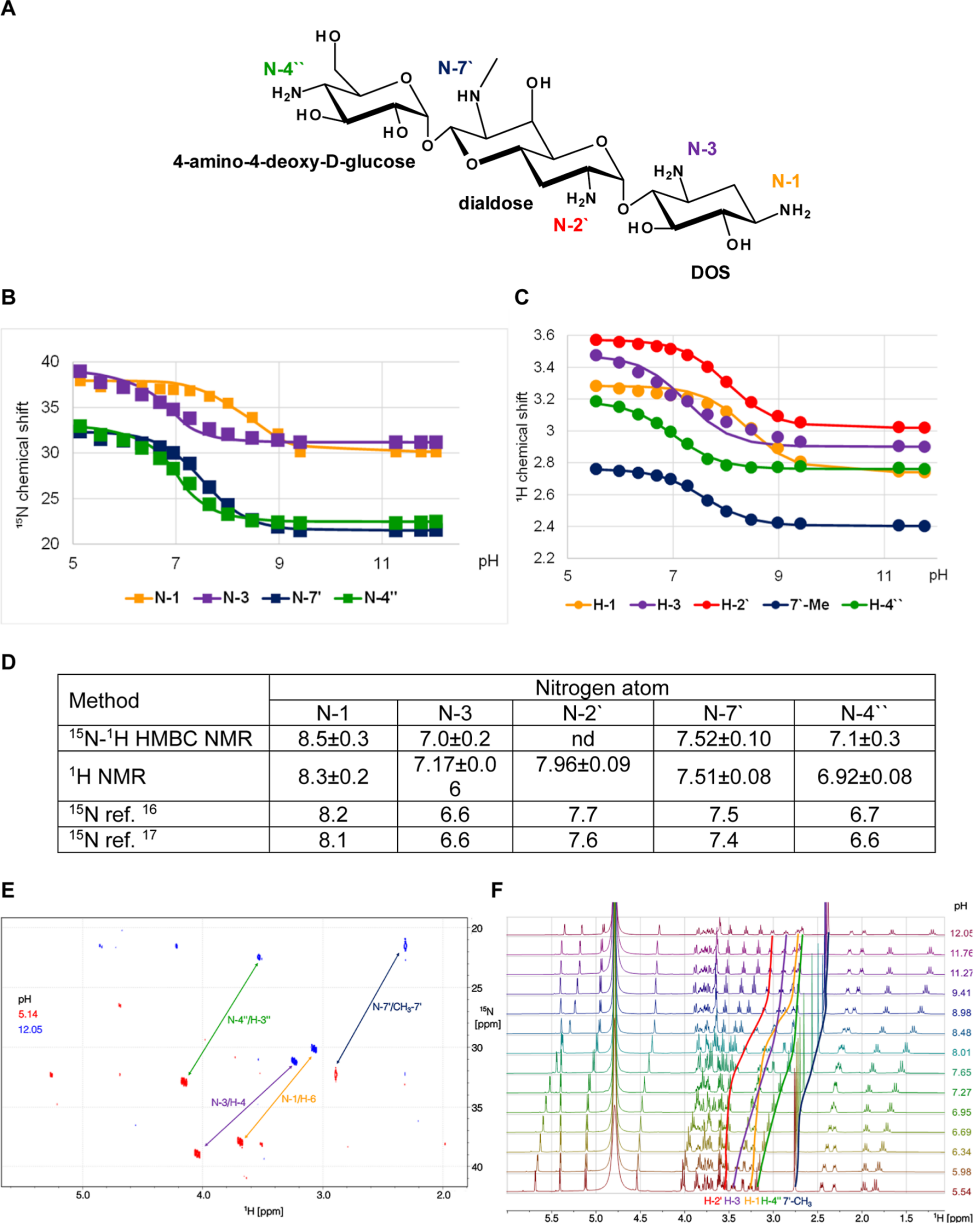
p*K*_a_ determination for the individual amino groups of apramycin. (A) Chemical structure and atom numbering of apramycin. (B) ^15^N NMR chemical shift titration curves. (C) ^1^H NMR chemical shift titration curves. (D) Determined p*K*_a_ values in comparison to values published previously. (E) ^15^N–^1^H HMBC spectra of apramycin in D_2_O. Arrows indicate the shift of signals upon pH change from 12.05 to 5.14. (F) Stacked ^1^H spectra of apramycin in D_2_O in the pH interval between 11.76 and 5.54.

Apramycin is a structurally unique aminoglycoside that comprises a 4-monosubstituted deoxystreptamine aminocyclitol moiety, an unusual bicyclic eight-carbon dialdose, and 4-amino-4-deoxy-d-glucose (Figure 6A). It has four primary amine groups, N-1 and N-3 on the 2-deoxystreptamine (DOS) fragment, N-2′ on the bicyclic eight-carbon dialdose, and N-4′′ on the terminal amino sugar moiety as well as one secondary amine (N-7′) on the central bicyclic sugar moiety. The N-2′ atom was not observed by ^15^N–^1^H HMBC NMR and its p*K*_a_ value was determined from the H-2′ chemical shift data (Figure 6B–E). For the other amines, the p*K*_a_ values determined using ^15^N and ^1^H chemical shifts are in agreement, taking into account the uncertainty. The order of the p*K*_a_ values is N-1 > N-2′ > N-7′ > N-3 ≈ N-4′′, which is consistent with literature data (Figure 6F).^16,17^ However, our determined p*K*_a_ values are higher by about 0.3 unit, except for N-7′ (the only secondary amine).

### p*K*_a_ Differences for Particular Amino Groups in Different Aminoglycosides

Our results confirm previous reports that have pointed to a variation in p*K*_a_ values of homologous amino groups from one aminoglycoside to another depending on their chemical environment. Thus, for example, the primary N-3′′ amines in the kanosamine series are less basic than the secondary N-3′′ amines in the garosamines. The basicity of N-6′ across the compounds studied is gentamicin ≈ arbekacin > tobramycin > amikacin > plazomicin. With the exception of plazomicin, this sequence is readily explained by the absence of electron-withdrawing and basicity-reducing C–O at the 3′ and 4′-positions in gentamicin and arbekacin, and the absence of a 3′–C–O bond in tobramycin, as compared to amikacin with the full complement of C–O bonds at the 3′ and 4′ positions. The absence of electron-withdrawing 3′- and 4′–C–O bonds in plazomicin is offset by the sp^2^-hybridized nature of C5′ and by the electron-withdrawing β–C–O bond in the hydroxyethyl moiety, which together make its N-6′ the least basic in the series. These trends are in full agreement with well-established patterns of p*K*_a_ modulation in simple amines.^27^ Finally, the greater basicity of N-3 in the N-1-γ-amino-α-hydroxybutyryl substituted aminoglycosides amikacin, arbekacin, and plazomicin arises because of the absence of a basic amino group at N-1, whose protonation in apramycin, tobramycin, and gentamicin reduces the basicity of N-3.

### Comparison of Protonation Profiles and Implications for Uptake of the Studied Aminoglycosides

**Figure 7:**
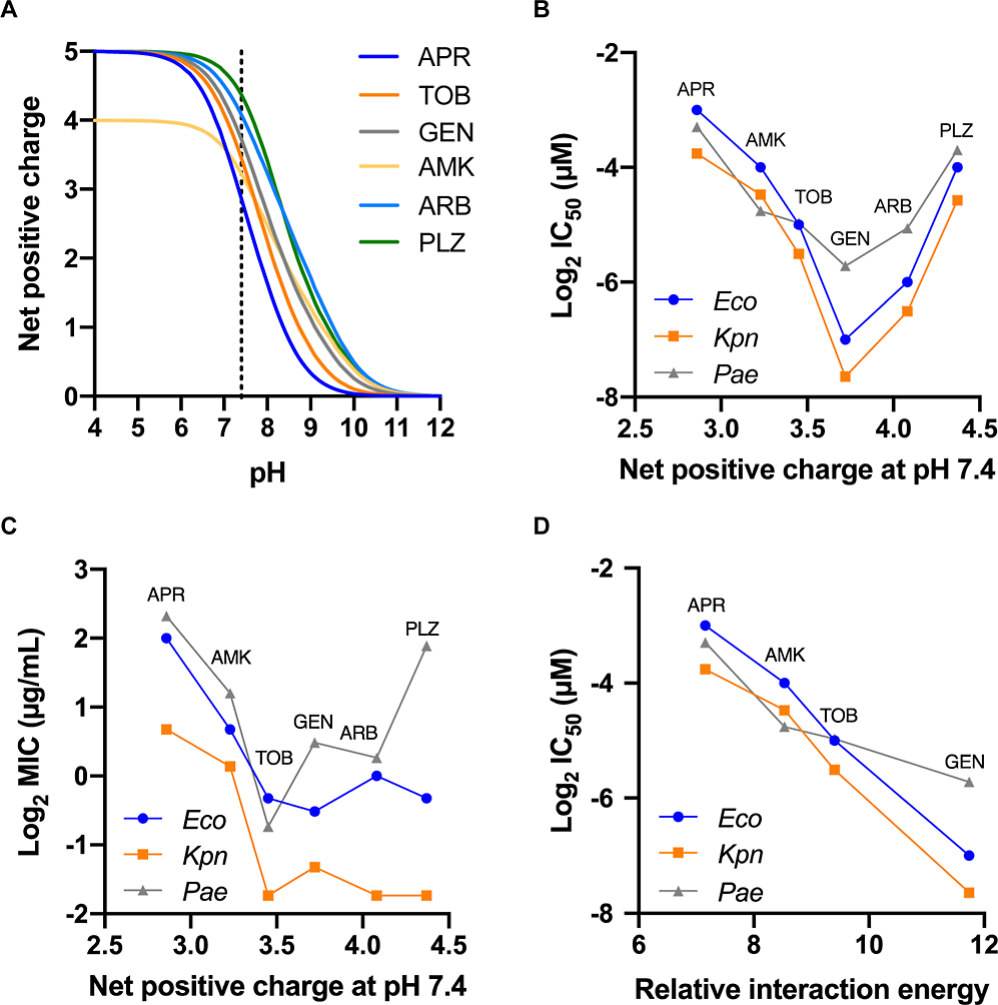
Effect of p*K*_a_ values on the net positive charge, inhibition of ribosomal translation, and bacterial inhibition. (A) Net positive charge of the studied aminoglycosides in the pH range 4–12 calculated using the Henderson–Hasselbalch equation from the determined p*K*_a_ values of individual amino groups. The dotted line indicates a pH of 7.4. (B) Relationship between the aminoglycoside net positive charge and ribosomal inhibition at physiologic pH. (C) Relationship between the net positive charge and cell-based bacterial inhibition expressed as the minimal inhibitory concentration (MIC), a function of both cellular uptake and ribosomal inhibition. (D) Relationship between the aminoglycoside relative interaction energy with rRNA (in kcal/mol, Table S7) and reported ribosomal inhibition at pH 7.4.

As summarized in Table 1, all of the determined p*K*_a_ values are between 6.4 and 9.8. This means that within a slightly wider pH range of approximately 5–11, the net charge of each aminoglycoside will depend on the exact p*K*_a_ values of its containing amino groups and the precise pH of the environment. We used the Henderson–Hasselbalch equation to calculate the net positive charges of the studied aminoglycosides in the pH range of 4–12 (Figure 7A). This showed that apramycin has the lowest charge at pH > 7, followed by tobramycin and gentamicin, whereas arbekacin and plazomicin have the highest charges. Amikacin is somewhat different because it contains only four amino groups; therefore, its calculated titration curve crosses the other ones, and it has the lowest positive charge of all tested aminoglycosides at pH < 7.

**Table 1:** Comparison of the Determined p*K*_a_ Values of Individual Amino Groups Among Apramycin, Tobramycin, Gentamicin, Amikacin, Arbekacin, and Plazomicin^a^

aminoglycoside	method	N-1	N-3	N-2′	N-6′	N-7′	N-4′′	N-3′′	N-4′′′
apramycin	^15^N–^1^H HMBC NMR	8.5 ± 0.3	7.0 ± 0.2	nd		7.52 ± 0.10	7.1 ± 0.3		
	^1^H NMR	8.3 ± 0.2	7.17 ± 0.06	7.96 ± 0.09		7.51 ± 0.08	6.92 ± 0.08		
tobramycin	^15^N–^1^H HMBC NMR	7.81 ± 0.06	6.93 ± 0.03	nd	8.90 ± 0.06			7.82 ± 0.08	
	^1^H NMR	7.61 ± 0.09	7.03 ± 0.12	8.07 ± 0.09	9.0 ± 0.3			7.75 ± 0.07	
gentamicin	^15^N–^1^H HMBC NMR	7.84 ± 0.10	7.15 ± 0.10	nd	9.2 ± 0.2			8.54 ± 0.07	
	^1^H NMR	nd	nd	nd	9.73 ± 0.16			8.53 ± 0.15	
amikacin	^15^N–^1^H HMBC NMR		7.53 ± 0.13		8.76 ± 0.13			7.96 ± 0.11	9.6 ± 0.4
	^1^H NMR		7.35 ± 0.14		8.7 ± 0.2			7.81 ± 0.16	9.8 ± 0.3
arbekacin	^15^N–^1^H HMBC NMR		7.39 ± 0.10	nd	nd			7.94 ± 0.07	9.71 ± 0.08
	^1^H NMR		7.26 ± 0.18	8.47 ± 0.19	9.11 ± 0.04			7.7 ± 0.2	9.8 ± 0.2
plazomicin	^15^N–^1^H HMBC NMR		7.84 ± 0.11	8.06 ± 0.08	8.52 ± 0.11			8.57 ± 0.08	9.74 ± 0.10
	^1^H NMR		7.8 ± 0.4	7.83 ± 0.14	8.43 ± 0.07			8.65 ± 0.05	nd

a nd-not determined.

The proton motive force has been established as the driving force for aminoglycoside uptake into bacterial cells,^28−30^ which in turn correlates with antibacterial activity.^31,32^ At physiological pH 7.4, the aminoglycoside net charge is between +2.86 e for apramycin and +4.37 e for plazomicin. Under these conditions, the pH difference between the environment (7.4) and bacterial cytoplasm (7.5) does not contribute effectively to the proton motive force and the electrical potential across the membrane provides the main stimulus for aminoglycoside uptake.^28^ Thus, at physiological pH plazomicin is expected to have a better uptake due to its higher net charge, followed by arbekacin (+4.08 e), gentamicin (+3.72 e), tobramycin (+3.45 e), amikacin (+3.23 e), and apramycin (+2.86 e). This prediction has yet to be tested experimentally by pH-dependent bacterial cell-wall permeability assays. In an acidic environment such as urine with a variable pH of e.g., 6.0, the proton motive force is significantly decreased due to lowered membrane potential.^33^ However, the net charge of all the aminoglycosides does not differ much from one another at pH 6.0 (+4.79 e for apramycin, +4.86 e for tobramycin, +4.90 e for gentamicin, 4.94 e for arbekacin, and 4.97 e for plazomicin), except for amikacin (+3.95 e), which has only four amino groups. Compared to pH 7.4, the increase in protonation level at pH 6.0 is the highest for apramycin (Δ = 1.93 e), followed by tobramycin (Δ = 1.41 e), gentamicin (Δ = 1.18 e), arbekacin (Δ = 0.86 e), amikacin (Δ = 0.72 e), and lowest for plazomicin (Δ = 0.60 e). A relatively larger increase in the protonation level at pH 6.0 may potentially make up for the reduced proton motive force, at least in part. Thus, apramycin is expected to show a comparable uptake to plazomicin and the other aminoglycosides at slightly acidic sites of infection, which has recently also been observed experimentally.^34^

### Aminoglycoside Net Charge–Activity Relationships

To investigate the relationship between the aminoglycoside p*K*_a_ values and antibacterial activity, we first compared their net charge at pH 7.4 with the reported cell-free ribosome inhibition levels (Figure 7B). In the series apramycin, amikacin, tobramycin, and gentamicin, the IC_50_ values clearly decrease (inhibition increases) with increased net positive charge. A higher protonation level as in arbekacin and plazomicin does not further enhance the inhibition, implying either a saturation in effect of positive charge or that the effect of additional protonation in arbekacin and plazomicin is offset by suboptimal fit into the binding pocket. Notably, the higher net positive charge for the latter two aminoglycosides is largely due to protonation of their γ-amino-α-hydroxybutyryl substituent with the high (9.7) p*K*_a_ of its amino group that is fully protonated at pH 7.4, suggesting that protonation of the N-4′′′ amino group in these compounds might not be important for ribosome inhibitory activity.

Next, we analyzed the correlation with cell-based minimal inhibitory concentration (MIC) values, which measure bacterial inhibition as a combination of both uptake and ribosome inhibition (Figure 7C). In the series apramycin, amikacin, and tobramycin, the MIC values decrease with an increased net positive charge, resembling the effect for ribosome inhibition alone. In this sequence, the net charge effect on ribosomal inhibition alone could be responsible for the lower MICs, although it cannot be ruled out that the effect on uptake is also contributing. In contrast, gentamicin, arbekacin, and plazomicin all show similar MIC values despite differences in their ribosomal IC_50_ values. This suggests that an increased uptake of plazomicin and arbekacin may potentially compensate for their reduced ribosomal IC_50_ values in comparison to gentamicin. Along similar lines, we have shown previously that simply increasing the number of amino groups in a given aminoglycoside does not always result in higher activity and may even lead to a reduction in activity. For example, replacement of the C-6′ hydroxy group of apramycin by an amino group resulted in a substantial loss of activity.^35^ Similarly, exchanging the 5′′-hydroxy group of the 4,5-disubstituted 2-deoxystreptamines for an amino group mostly leads to a reduction in activity because of the proximity of the newly introduced basic residue with the 2′-ammonium ion.^36^

### Structure—p*K*_a_—rRNA Binding Relationships

As described above, the aminoglycosides contain primary amine groups either directly attached to the sugar ring or linked through a methylene bridge (aminomethyl group) or through an α-hydroxybutyryl chain as well as secondary amine groups (*N*-methyl and N-2-hydroxyethyl groups). The lowest p*K*_a_ values were consistently found for the N-3 and N-1 primary amino groups on the 4,6-disubstituted DOS moiety. The highest p*K*_a_s were for the primary 1-aminoalkyl groups (γ-amino-α-hydroxybutyryl and aminomethyl groups), followed by the secondary amines. Thereby, the substitution of the N-1 amino group with an l-γ-amino-α-hydroxybutyryl group in amikacin, arbekacin, and plazomicin results in a significant increase in the basicity of the aminoglycoside. Similarly, the addition of a methyl substituent to the primary N-3′′ amino group in gentamicin and plazomicin increases its basicity. In contrast, the introduction of a 2-hydroxyethyl group in the aminomethyl moiety (N-6′) of plazomicin decreases its basicity.

**Figure 8:**
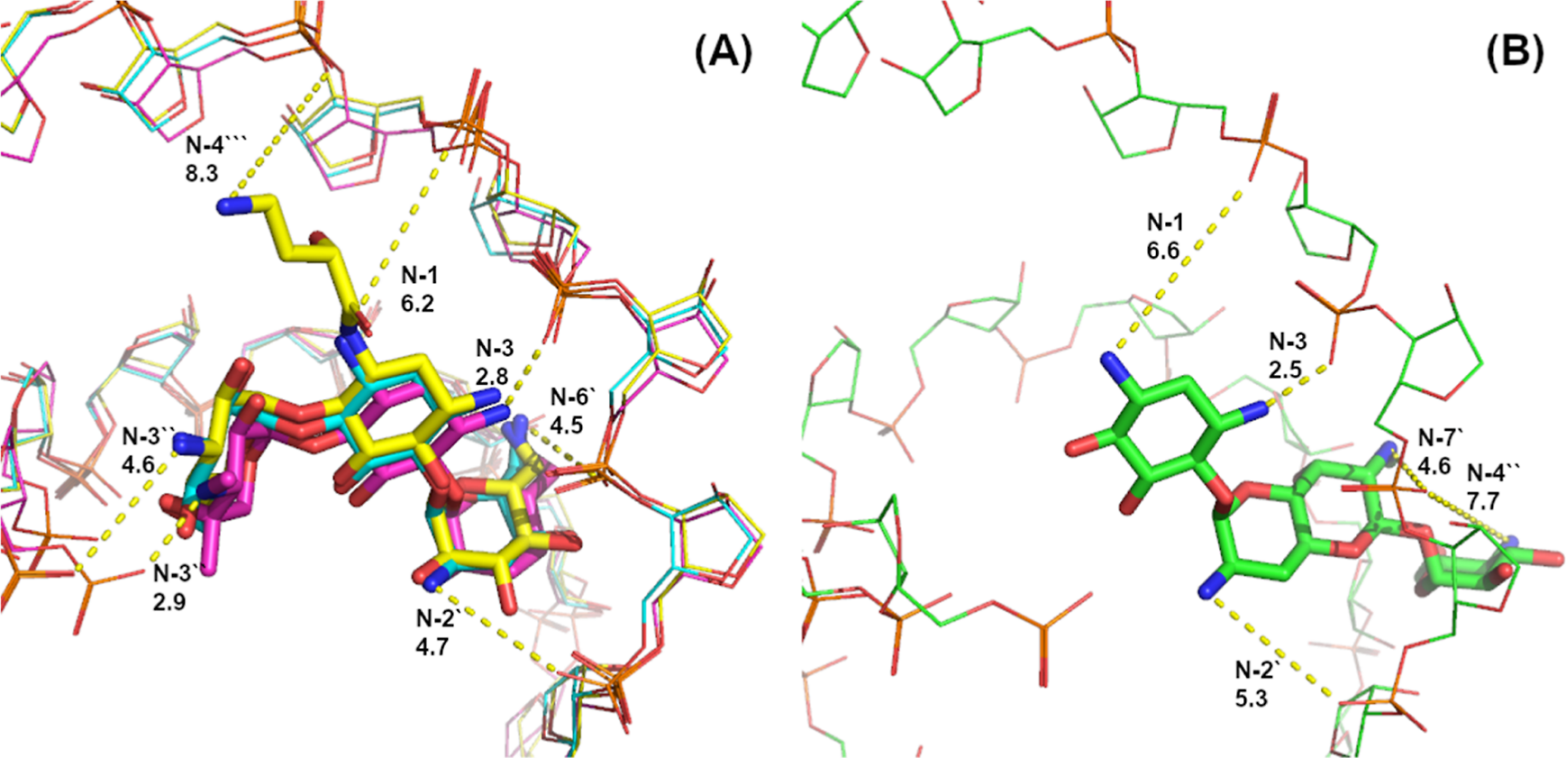
(A) Superposition of the crystal structures of tobramycin (cyan, PDB ID 1LC4(44)), gentamicin (purple, 2ET3(45)) and amikacin (yellow, 4P20(46)) bound with rRNA. (B) Crystal structure of apramycin (green, PDB ID 4AQY(4)) bound with rRNA. The aminoglycosides are shown in stick representation and the rRNA backbone is shown with thin lines. The N, O, and P atoms are colored in blue, red, and orange, respectively. The distances between amino groups and nearest phosphates are marked with yellow dashed lines and are indicated in Å.

To evaluate the potential impact of the p*K*_a_ values on the binding strength with rRNA (i.e., binding free energy, Δ*G*), we analyzed the crystal structures of apramycin, tobramycin, gentamicin, and amikacin bound to the A-site of 16S rRNA available from the Protein Data Bank. As shown in Figure 8A, tobramycin, gentamicin, and amikacin bind rRNA in nearly identical conformation. The DOS moiety of apramycin interacts with rRNA very similarly to and overlays with the other aminoglycosides, except for its additional 4-amino-4-deoxy-d-glucosyl residue (Figure 8B).^37^

In general, the aminoglycosides interact with the rRNA through specific hydrogen bonding with the nucleotide bases and by nonspecific charge interactions with the rRNA backbone phosphate groups. Since the protonation state of the aminoglycoside amines is primarily expected to modulate the charge interactions, we only consider the latter, i.e., amine–phosphate interactions, in our analysis. The shortest distance from an amine to an rRNA phosphate group in all the complexes is measured from the N-3 amine (about 2.5–2.8 Å), which also has the lowest p*K*_a_ value (Table 1). Short distance amine–phosphate interactions are also observed with the N-3′′ (about 2.9 Å in gentamicin and 4.6 Å in tobramycin and amikacin), N-6′/N-7′ (4.5–4.6 Å), and N-2′ (4.7–5.3 Å) amino groups. The N-1, N-4′′ (in apramycin), and N-4′′′ (in amikacin) amino groups are >6 Å away from any phosphates. We calculated the relative electrostatic interaction energies for each amino group at pH 7.4 and pH 6.0 by considering interactions with only the closest phosphate group (Table S7). The sum of these energies shows a strong relationship with ribosomal IC_50_ values, indicating that the electrostatic interactions are primarily responsible for binding strength, whereas the hydrogen bonding with bases determines specificity (Figure 7D). At physiological pH, the N-6′ amino group contributes most to the binding of tobramycin and amikacin, while for gentamicin, the N-3′′ amine shows the highest relative interaction energy (due to a shorter distance and an increased *N*-methyl p*K*_a_ value), followed by N-6′. For apramycin, the N-2′ and N-1 amines show comparable relative interaction energies owing to their elevated p*K*_a_ values. This suggested that combining the central DOS moiety with the N-6′ amine-containing nebrosamine from tobramycin and the N-3′′-containing garosamine from gentamicin (Figure S1) could lead to an aminoglycoside derivative with increased binding affinity. Indeed, the literature reveals that this compound was previously prepared by Mallams and co-workers^38^ and was found to have excellent activity when tested against *Escherichia coli* and *Pseudomonas aeruginosa*.^39^ At pH 6.0, the N-3 amino group largely determines the binding strength between the aminoglycosides and rRNA in a p*K*_a_-dependent manner (highest relative energy for apramycin, then amikacin, gentamicin, and lowest for tobramycin). The protonation levels of the other amines of tobramycin, gentamicin, and amikacin probably have a much smaller effect on the binding strength due to larger distances to phosphates and because their p*K*_a_s suggest nearly full protonation already at physiological pH. In line with the net charge–ribosomal IC_50_ relationships, the introduction of the l-γ-amino-α-hydroxybutyryl group in amikacin does not seem to affect the binding strength with rRNA as both, the N-1 and N-4′′′ amino groups, are >6 Å away from any phosphates. Furthermore, it has previously been suggested that amine protonation not only facilitates electrostatic interaction with the negatively charged phosphate backbone but also affects intramolecular charge repulsion and hydrogen bonding that modulate the conformation and flexibility of an aminoglycoside antibiotic, which may in turn determine its specific affinity to the three-dimensional RNA binding pocket, and thus, its biological activity.^40,41^

## Conclusions

We used ^15^N–^1^H HMBC and ^1^H NMR spectroscopy to determine the p*K*_a_ values of individual amino groups in the aminoglycosides apramycin, tobramycin, gentamicin, amikacin, arbekacin, and plazomicin. The values for arbekacin and plazomicin are reported for the first time. Although ^1^H NMR is incomparably faster and more sensitive, the signal overlap observed in several cases causes difficulties in following the chemical shift changes, thereby not allowing precise determination of the p*K*_a_ values. ^15^N–^1^H HMBC experiments, on the other hand, may miss signals due to low sensitivity. Thus, a combination of the two experimental approaches is pertinent for the determination of all of the aminoglycoside amine p*K*_a_ values.

The amine p*K*_a_ values allow for calculation of the net charge that is important for understanding the antibacterial activity and uptake of the aminoglycosides under various pH conditions. The individual amine p*K*_a_ values obtained using the same methodology/approach also allow for a direct comparison of the aminoglycoside amino groups in terms of protonation levels at a particular pH, which are important to understand their binding mechanism with rRNA. Thus, we have shown that the N-6′ amino group of tobramycin and amikacin, the N-3′′ and N-6′ amines of gentamicin, and the N-2′ and N-1 amines of apramycin are most important for binding to their drug target. The N-4′′′ amine of the l-γ-amino-α-hydroxybutyryl group does not seem to affect the binding strength with rRNA and does not enhance the ribosomal inhibition of arbekacin and plazomicin. We expect the approach described herein to be valuable for rationalization of the structure–activity relationships of aminoglycosides and for the design of better antibiotics.

## Methods

### Materials and General Methods

Deuterium oxide, deuterium chloride solution (35 wt % in D_2_O, ≥99 atom % D), sodium deuteroxide solution (40 wt % in D_2_O, 99.5 atom % D), tobramycin, gentamicin, amikacin, and apramycin were purchased from Sigma-Aldrich. Plazomicin (Cipla) and arbekacin (Meiji Seika) solutions were obtained from the dispensary.

All NMR spectra, including ^1^H, ^13^C, HSQC, HMBC, COSY, and ^1^H–^15^N HMBC were recorded on a Bruker Avance Neo 600 MHz (Bruker Biospin Gmbh, Rheinstetten, Germany) with a quadruple resonance CryoProbe (CP QCI 600S3 H/F-C/N-D-05 Z).

### Titration Experiments

The initial concentrations of the aminoglycosides were from 0.3 to 0.6 mol/L and during the titration process, the concentration could be decreased up to 2 times.

Each aminoglycoside was titrated three times using the deuterium chloride and sodium deuteroxide solutions to obtain the desired pH value. The pD values were measured using an Oakton pH 11 Portable pH/mV/°C Meter and then converted into pH (pH = pD – 0.5) following the IUPAC Technical Report Guidelines.^21,42^

Before the pH measurements, the electrode was calibrated using three points (standard buffer solutions with pH values 4, 7, and 10).

The p*K*_a_ was calculated using MS Excel Solver Add-in to align the experimental titration graph with the predicted values using the formula
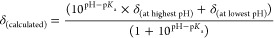


The MS Solver can be used for nonlinear calculation of the different values with the same error level as MATLAB or OriginPro.^43^ The standard deviation was calculated from the three obtained values.

### Calculation of Relative Electrostatic Interaction Energies

The relative electrostatic interaction energies were calculated using the Coulomb’s equation , where *k* is an arbitrary scaling constant (1000), *q*_1_ is the p*K*_a_-dependent charge (protonation level) of an aminoglycoside amino group at a particular pH value calculated using the Henderson–Hasselbalch equation, *q*_2_ is the charge of an rRNA backbone phosphate (fully deprotonated at pH 6.0–7.4, resulting in −1 e in all cases), *D* is the dielectric constant of water (80), and *r* is the distance between the amino group nitrogen and the phosphate oxygen in the aminoglycoside-bound rRNA crystal structure.

### Calculation of Net Charges

The net positive charge of all the aminoglycosides in the pH range 4–12 were calculated using the equation  derived from the Henderson–Hasselbalch equation, where p*K*_a_i__ is the average p*K*_a_ value of each amino group measured by ^15^N–^1^H HMBC and ^1^H NMR experiments.

### Ribosomal Inhibition Assays

Cell-free *in vitro* translation inhibition assays were performed as described previously using bacterial S30 extracts and luciferase mRNA.^23^ In brief, firefly luciferase mRNA was transcribed *in vitro* using a T7 RNA polymerase. Each reaction comprised bacterial S30 extract, 0.2 mM amino acid mix, 6 μg of tRNA, 0.4 μg of hFluc mRNA, 0.3 μL of protease inhibitor, 12 U of RNase inhibitor, and 6 μL of S30 premix without amino acids, plus a defined concentration of aminoglycoside antibiotic. After 1 h of incubation at 37 °C, the reaction was stopped on ice, 75 μL of luciferase assay reagent was added to each well, and the luminescence recorded.

### Bacterial Inhibition Assays

The MICs of aminoglycosides for *E. coli* strain ATCC 25922, *Klebsiella pneumoniae* clinical isolate AG215, and *P. aeruginosa* strain ATCC 27853 were determined by broth microdilution assays according to CLSI reference methodology M07 and as described previously.^5^ The MIC values plotted represent the geometric mean of *n* ≥ 3 replicate values for each aminoglycoside.

## Supporting Information Available

^1^H and ^13^C NMR chemical shift assignments for all compounds and chemical structure of an aminoglycoside derivative combining the central DOS moiety with the N-6′ amine-containing nebrosamine from tobramycin and the N-3′-containing garosamine from gentamicin (PDF)
