# Supplementary material for: Experimental Determination of the pKa Values of Clinically Relevant Aminoglycoside Antibiotics: Toward Establishing pKa—Activity Relationships
Source: ACS Omega. 2024 Jan 26;9(5):5876–87. doi: 10.1021/acsomega.3c09226 (PMC10851411; doi:10.1021/acsomega.3c09226)
Supplement: Supplementary file 1 — ao3c09226_si_001.pdf [file ao3c09226_si_001.pdf]

# Experimental determination of the p$K_a$ values of clinically relevant aminoglycoside antibiotics: towards establishing p$K_a$ – activity relationships

Ruslans Muhamadejevs, Klara Haldimann, Marina Gysin, David Crich, Kristaps Jaudzems*, Sven N. Hobbie*

## Supporting Information

**Table S1.** $^{1}H$ and $^{13}C$ NMR chemical shift assignments for apramycin at pH 5.14.

| Atom number | δ $^{1}H$, ppm | δ $^{13}C$, ppm |
|---|---|---|
| 1 | 3.43 | 49.89 |
| 2 | 1.99, 2.59 | 28.41 |
| 3 | 3.63 | 48.65 |
| 4 | 4.04 | 78.06 |
| 5 | 3.77 | 75.10 |
| 6 | 3.69 | 72.53 |
| 1` | 5.83 | 95.23 |
| 2` | 3.74 | 48.03 |
| 3` | 2.12, 2.46 | 26.98 |
| 4` | 3.99 | 66.08 |
| 5` | 3.87 | 69.59 |
| 6` | 4.69 | 62.69 |
| 7` | 3.51 | 59.38 |
| 7`-NH-**$CH_3$** | 2.89 | 30.31 |
| 8` | 5.26 | 92.82 |
| 1`` | 5.56 | 94.52 |
| 2`` | 3.77 | 70.39 |
| 3`` | 4.14 | 69.43 |
| 4`` | 3.36 | 52.19 |
| 5`` | 4.16 | 68.22 |
| 6`` | 3.87, 3.93 | 60.41 |

**Table S2.** $^{1}H$ and $^{13}C$ NMR chemical shift assignments for tobramycin at pH 11.60.

| Atom number | δ $^{1}H$, ppm | δ $^{13}C$, ppm |
|---|---|---|
| 1 | 2.97 | 50.44 |
| 2 | 1.30, 2.03 | 35.57 |
| 3 | 2.93 | 49.52 |
| 4 | 3.39 | 86.66 |
| 5 | 3.70 | 74.45 |
| 6 | 3.32 | 88.15 |
| 1` | 5.21 | 99.80 |
| 2` | 3.01 | 49.25 |
| 3` | 1.67, 2.09 | 35.02 |
| 4` | 3.59 | 66.28 |
| 5` | 3.66 | 73.97 |
| 6` | 2.78, 3.04 | 41.75 |
| 1`` | 5.12 | 100.02 |
| 2`` | 3.57 | 71.91 |
| 3`` | 3.06 | 54.34 |
| 4`` | 3.39 | 69.43 |
| 5`` | 3.98 | 72.20 |
| 6`` | 3.83 | 60.41 |

**Table S3.** $^{1}H$ and $^{13}C$ NMR chemical shift assignments for gentamicin at pH 11.68.

| Atom number | δ $^{1}H$, ppm | δ $^{13}C$, ppm |
|---|---|---|
| 1 | 2.90 | 50.80 |
| 2 | 1.25, 1.49 | 35.63 |
| 3 | 2.90 | 49.75-49.97 |
| 4 | 3.34 | 87.27-87.77 |
| 5 | 3.62 | 74.40 |
| 6 | 3.29 | 86.73 |
| 1` | 5.18 | 101.35 |
| 2` | 2.90 | 49.75-49.97 |
| 3` | 1.65-1.80 | 25.14-26.25 |
| 4` | 1.49-1.80 | 25.14-27.56 |
| 5` | 3.62-3.82 | 72.17-73.76 |
| 6` | 2.67-2.90 | 45.10-57.18 |
| 6`-$CH_3$ | 1.09 | 14.12-18.06 |
| 6`-NH-**$CH_3$** | 2.32 | 32.69 |
| 1`` | 5.13 | 100.49 |
| 2`` | 3.82 | 69.37 |
| 3`` | 2.59 | 63.39 |
| 3``-NH-**$CH_3$** | 2.55 | 37.12 |
| 4`` | 3.88 | 72.49 |
| 4``-**$CH_3$** | 1.25 | 21.75 |
| 5`` | 3.34 ,4.08 | 67.75 |

**Table S4.** $^1$H and $^{13}$C NMR chemical shift assignments for amikacin at pH 11.71.

| Atom number | δ $^1$H, ppm | δ $^{13}$C, ppm |
|---|---|---|
| 1 | 3.99 | 49.60 |
| 2 | 1.43, 1.97 | 34.25 |
| 3 | 2.94 | 48.60 |
| 4 | 3.34 | 87.10 |
| 5 | 3.74 | 74.49 |
| 6 | 3.74 | 80.21 |
| 1` | 5.32 | 99.95 |
| 2` | 3.61 | 72.06 |
| 3` | 3.74 | 73.15 |
| 4` | 3.34 | 71.06 |
| 5` | 3.78 | 73.10 |
| 6` | 2.76, 3.00 | 41.58 |
| 1`` | 5.09 | 98.23 |
| 2`` | 3.39 | 71.64 |
| 3`` | 3.00 | 54.14 |
| 4`` | 3.34 | 69.27 |
| 5`` | 3.99 | 72.03 |
| 6`` | 3.78 | 60.31 |
| 1``` | - | 176.89 |
| 2``` | 4.19 | 69.94 |
| 3``` | 1.74, 1.91 | 36.48 |
| 4``` | 2.76 | 37.35 |

**Table S5.** $^{1}H$ and $^{13}C$ NMR chemical shift assignments for amikacin at pH 4.60.

| Atom number | δ $^{1}H$, ppm | δ $^{13}C$, ppm |
|---|---|---|
| 1 | 4.24 | 49.04 |
| 2 | 2.02, 2.35 | 30.40 |
| 3 | 3.65 | 48.82 |
| 4 | 4.14 | 77.11 |
| 5 | 3.93 | 74.85 |
| 6 | 4.00 | 79.99 |
| 1\` | 5.95 | 94.60 |
| 2\` | 3.69 | 48.72 |
| 3\` | 2.14, 2.20 | 20.64 |
| 4\` | 1.72, 2.07 | 25.55 |
| 5\` | 4.30 | 66.05 |
| 6\` | 3.22, 3.38 | 42.78 |
| 1\`\` | 5.30 | 97.98 |
| 2\`\` | 3.93 | 67.98 |
| 3\`\` | 3.56 | 55.26 |
| 4\`\` | 3.81 | 65.64 |
| 5\`\` | 4.17 | 72.05 |
| 6\`\` | 3.93 | 59.84 |
| 1\`\`\` | - | 175.45 |
| 2\`\`\` | 4.41 | 69.64 |
| 3\`\`\` | 2.11, 2.28 | 30.90 |
| 4\`\`\` | 3.29 | 37.09 |

**Table S6.** $^{1}H$ and $^{13}C$ NMR chemical shift assignments for plazomicin at pH 12.19.

| Atom number | δ $^{1}H$, ppm | δ $^{13}C$, ppm |
|---|---|---|
| 1 | 3.92 | 49.55 |
| 2 | 1.43, 1.91 | 34.31 |
| 3 | 2.78 | 49.41 |
| 4 | 3.45 | 83.89 |
| 5 | 3.62 | 74.73 |
| 6 | 3.71 | 79.59 |
| 1` | 5.32 | 100.07 |
| 2` | 3.07 | 46.49 |
| 3` | 1.98, 2.19 | 24.79 |
| 4` | 4.88 | 98.39 |
| 5` | - | 146.61 |
| 6` | 3.14 | 49.94 |
| 7` | 2.68 | 49.26 |
| 8` | 3.64 | 60.23 |
| 1`` | 5.08 | 98.30 |
| 2`` | 3.66 | 69.29 |
| 3`` | 2.50 | 63.49 |
| N-3``-$CH_3$ | 2.46 | 37.13 |
| 4`` | - | 72.32 |
| 4``-CH3 | 1.18 | 21.79 |
| 5`` | 3.26, 4.05 | 67.79 |
| 1``` | - | 179.07 |
| 2``` | 4.09 | 68.79 |
| 3``` | 1.64, 1.82 | 37.67 |
| 4``` | 2.68 | 37.67 |

**Table S7.** Relative energies in kcal/mol for the closest amine-phosphate electrostatic interactions as observed in the crystal structures of apramycin, tobramycin, gentamicin and amikacin rRNA complexes with PDB IDs 4AQY, 1LC4, 2ET3, 4P20. Higher numbers mean stronger interaction.

| Aminoglycoside | pH | N-1 | N-3 | N-2` | N-6` | N-7` | N-4`` | N-3`` | N-4``` |
|---|---|---|---|---|---|---|---|---|---|
| Apramycin | 7.4 | 1.72 | 1.58 | 1.85 | - | 1.54 | 0.47 | - | - |
| | 6.0 | 1.89 | 4.60 | 2.33 | - | 2.64 | 1.48 | - | - |
| Tobramycin | 7.4 | 1.35 | 1.23 | 2.19 | 2.70 | - | - | 1.92 | - |
| | 6.0 | 1.98 | 4.04 | 2.64 | 2.77 | - | - | 2.67 | - |
| Gentamicin | 7.4 | 1.48 | 1.61 | 1.88 | 2.75 | - | - | 4.02 | - |
| | 6.0 | 1.99 | 4.17 | 2.62 | 2.78 | - | - | 4.30 | - |
| Amikacin | 7.4 | - | 2.33 | - | 2.65 | - | - | 2.05 | 1.50 |
| | 6.0 | - | 4.31 | - | 2.77 | - | - | 2.68 | 1.51 |

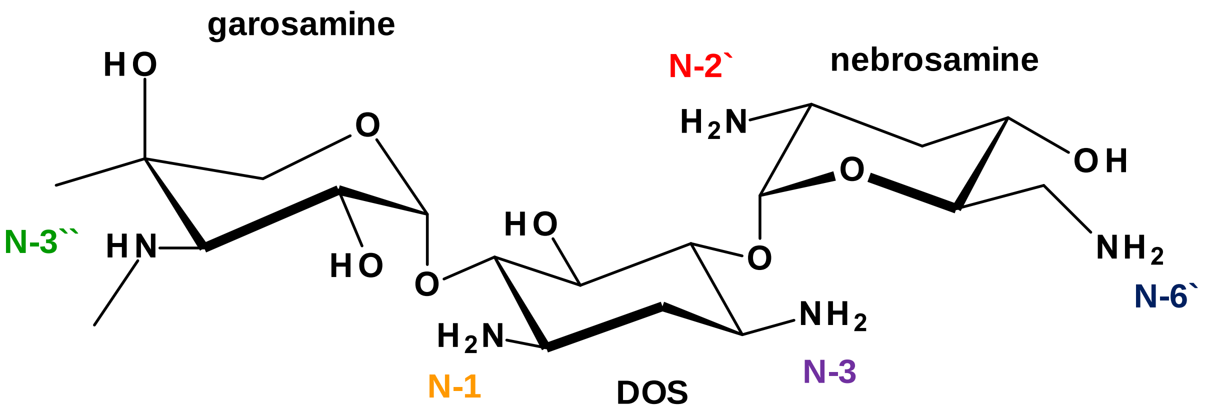


**Fig. S1.** Aminoglycoside derivate combining the central DOS moiety with the N-6` amine-containing nebrosamine from tobramycin and the N-3``-containing garosamine from gentamicin, a derivate that has previously been described.[1]

## References

(1) Kugelman, M.; Mallams, A. K.; Vernay, H. F. Semisynthetic Aminoglycoside Antibacterials. Part IV. Synthesis of Antibiotic JI-20A, Gentamicin B, and Related Compounds. J.C.S. Perkin I 1976, (10), 1126-1134.
